# Comparative Augmentative Efficacy of Second-Generation Antipsychotics in Adult Treatment-Resistant Depression: A Systematic Review

**DOI:** 10.7759/cureus.109712

**Published:** 2026-05-26

**Authors:** Rashed Khalid, Gihad Elbashir, Ahmed Hassan, Mayada Abdulbagi

**Affiliations:** 1 Psychiatry, Leeds and York Partnership NHS Foundation Trust, Leeds, GBR; 2 Psychiatry, Lancashire and South Cumbria NHS Foundation Trust, Preston, GBR; 3 General Adult Psychiatry, Mayo Mental Health Services, Castlebar, IRL; 4 Psychiatry, Berkshire Healthcare NHS Foundation Trust, Bracknell, GBR

**Keywords:** akathisia, aripiprazole, augmentation therapy, brexpiprazole, cariprazine, major depressive disorder, quetiapine, second-generation antipsychotics, systematic review, treatment-resistant depression

## Abstract

Treatment-resistant depression (TRD) affects a substantial proportion of patients with major depressive disorder and remains a major therapeutic challenge. Augmentation with second-generation antipsychotics (SGAs) is commonly used; however, comparative evidence regarding their efficacy and safety remains limited. This systematic review aimed to evaluate the augmentative efficacy and tolerability of SGAs in adults with TRD. A systematic search of PubMed/MEDLINE, Embase, Web of Science, Scopus, and the Cochrane Library was conducted for randomized controlled trials published between 2016 and 2025. Eligible studies included adults with TRD receiving SGA augmentation. Two reviewers independently screened studies, extracted data, and assessed risk of bias using the Cochrane RoB 2 tool. Due to clinical heterogeneity across interventions and outcomes, findings were synthesized narratively. Eleven randomized controlled trials including 5,300 participants were analyzed, evaluating brexpiprazole, cariprazine, aripiprazole, and quetiapine XR. Most studies reported greater reductions in depressive symptoms compared with placebo, commonly measured by changes in the Montgomery-Åsberg Depression Rating Scale. Brexpiprazole and the aripiprazole-sertraline combination showed the most consistent improvements, with brexpiprazole augmentation yielding remission rates ranging from 22 to 31% and cariprazine augmentation showing rates of 22-32% across flexible-dose trials. Several agents demonstrated early onset of action within 2-3 weeks. Common adverse events included akathisia, insomnia, restlessness, and weight gain. Discontinuation due to adverse events ranged from 0.4% to 8.6%. Ten studies were assessed as having a low risk of bias. SGA augmentation is an effective treatment strategy for TRD, with brexpiprazole showing particularly consistent benefits. Careful risk-benefit assessment and monitoring for adverse effects are essential. Further head-to-head and long-term studies are needed to guide personalized treatment.

## Introduction and background

Major depressive disorder (MDD) is a leading cause of disability worldwide, imposing a substantial burden on individuals, healthcare systems, and societies [1]. Although first-line antidepressant therapies are effective for many patients, a significant proportion fail to achieve adequate symptom remission [2]. Treatment-resistant depression (TRD), commonly defined as an insufficient response to at least two antidepressants administered at adequate doses and duration, affects approximately 10% of patients with MDD and is associated with poorer functional outcomes, increased suicide risk, and higher healthcare utilization [3].

In patients with TRD, pharmacological augmentation strategies are frequently employed to enhance antidepressant response [4]. Among these, second-generation antipsychotics (SGAs) have emerged as one of the most extensively studied and clinically utilized augmentation options [5]. Agents such as aripiprazole, quetiapine, olanzapine, brexpiprazole, and risperidone have demonstrated efficacy when added to ongoing antidepressant treatment, leading to their inclusion in several international treatment guidelines [6]. The antidepressant effects of SGAs are thought to arise from their complex pharmacodynamic profiles, meaning how these drugs interact with multiple receptors in the brain, including modulation of dopaminergic, serotonergic, and noradrenergic pathways, which are brain chemical systems implicated in mood regulation [7].

Despite their widespread use, SGAs differ substantially in receptor binding affinities, pharmacokinetics (how the body absorbs, distributes, and eliminates a drug), tolerability profiles, and adverse effect risks, including metabolic disturbances, extrapyramidal symptoms (movement disorders such as muscle stiffness or restlessness), sedation, and weight gain [8]. These differences are clinically meaningful and may influence both treatment selection and long-term adherence. While multiple randomized controlled trials (RCTs) and meta-analyses have evaluated the efficacy of individual SGAs as augmentation agents in TRD, direct comparative evidence across different SGAs remains limited and fragmented [9]. Consequently, clinicians often face challenges in choosing the most appropriate augmentation agent based on a balanced consideration of efficacy and safety.

Previous reviews have primarily focused on the overall effectiveness of antipsychotic augmentation as a class or on single-agent efficacy, with less emphasis on head-to-head or comparative outcomes. Furthermore, emerging data on newer SGAs, such as brexpiprazole and cariprazine, warrant updated synthesis to inform evidence-based decision-making. A systematic comparison of efficacy and safety outcomes across SGAs is therefore needed to clarify their relative benefits and risks in adult patients with TRD.

The present systematic review aims to comprehensively evaluate the comparative augmentative efficacy of SGAs in adults with TRD. Specific objectives include assessing the safety profile and long-term tolerability of these agents. By synthesizing available clinical evidence, this review seeks to support clinicians in optimizing augmentation strategies and to identify gaps for future research in the management of TRD.

## Review

Methodology

Study Design and Reporting Standards

This systematic review was conducted in accordance with the Preferred Reporting Items for Systematic Reviews and Meta-Analyses (PRISMA) 2020 guidelines [10]. The review methodology was prospectively designed to ensure transparency, reproducibility, and methodological rigor in the identification, selection, appraisal, and synthesis of relevant studies evaluating SGAs as augmentation therapy in adult TRD.

Information Sources and Search Strategy

A comprehensive and systematic literature search was performed across multiple electronic databases, including PubMed/MEDLINE, Embase, Web of Science, Scopus, and the Cochrane Library. These databases were selected to ensure broad coverage of biomedical, clinical, and pharmacological literature. The search strategy combined controlled vocabulary terms (e.g., MeSH and Emtree terms) and free-text keywords related to treatment-resistant depression, second-generation antipsychotics, and augmentation therapy. Boolean operators (“AND,” “OR”) were used to optimize sensitivity and specificity. To ensure inclusion of the most current and clinically relevant evidence, the search was restricted to studies published between January 2016 and December 2025. Reference lists of included studies and relevant reviews were also manually screened to identify additional eligible articles. The exact search strategy for each database is provided in the appendices.

Eligibility Criteria (PICOS Framework)

Eligibility criteria were defined a priori using the PICOS (Population, Intervention, Comparator, Outcomes, Study design) framework. The population of interest included adult patients (≥18 years) diagnosed with treatment-resistant MDD, typically defined as inadequate response to at least two antidepressant trials (ADTs) of adequate dose and duration. The intervention comprised SGAs used as augmentation therapy, including but not limited to aripiprazole, quetiapine, olanzapine, risperidone, brexpiprazole, and cariprazine, administered alongside ongoing antidepressant treatment. Comparators included placebo augmentation, antidepressant monotherapy, or alternative pharmacological augmentation strategies. Eligible outcomes included changes in validated depression rating scales, response and remission rates, and safety or tolerability outcomes such as adverse events and treatment discontinuation. Only RCTs and well-designed comparative clinical studies published in peer-reviewed journals were included, while case reports, narrative reviews, editorials, conference abstracts, and non-human studies were excluded.

Study Selection

All retrieved records were imported into EndNote X21 reference management software, where duplicate citations were identified and removed. Following deduplication, titles and abstracts were independently screened for relevance based on the predefined eligibility criteria. Full-text articles were subsequently assessed for inclusion. Discrepancies during the screening and selection process were resolved through discussion and consensus to minimize selection bias.

Data Extraction and Data Items

Data extraction was conducted using a standardized data extraction framework to ensure consistency across studies. Extracted information included study characteristics (author, year, country, study design), participant demographics, diagnostic criteria for treatment-resistant depression, intervention details (type and dose of antipsychotic), comparator characteristics, outcome measures related to efficacy and safety, and duration of follow-up. Where necessary, corresponding authors were consulted for clarification of missing or ambiguous data.

Risk of Bias Assessment

The methodological quality and risk of bias of included RCTs were assessed using the Cochrane Risk of Bias 2 (RoB 2) Tool [11]. This tool evaluates bias across key domains, including the randomization process, deviations from intended interventions, missing outcome data, measurement of outcomes, and selection of reported results. Each study was classified as having low risk, some concerns, or high risk of bias, and these assessments were incorporated into the interpretation of the findings.

Data Synthesis and Rationale for Non-Meta-Analysis

A narrative synthesis of the findings was undertaken due to substantial heterogeneity across the included studies. Considerable variability was observed in study designs, definitions of TRD, types and dosages of SGAs, duration of treatment, outcome measures, and reporting of efficacy endpoints. Additionally, differences in depression rating scales and inconsistent reporting of statistical data limited the feasibility of meaningful quantitative pooling. Conducting a meta-analysis under these conditions was deemed inappropriate, as it could yield misleading or clinically non-informative results. Instead, a structured qualitative comparison was performed to highlight patterns of efficacy and safety across different SGAs, allowing for a clinically relevant and methodologically sound synthesis of the available evidence.

Results

Study Selection Process

**Figure 1:**
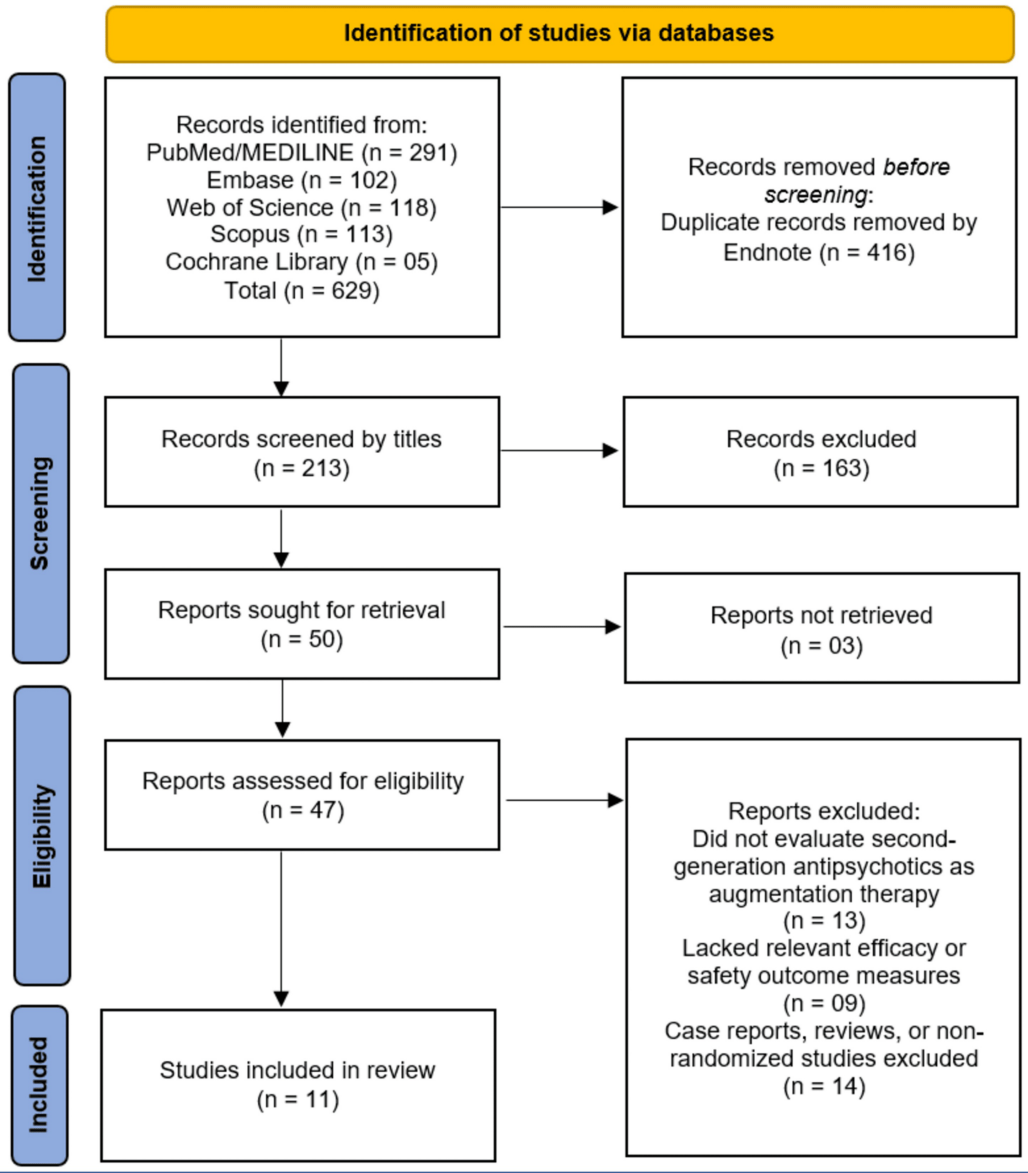
Study Selection Process

The systematic search of electronic databases (PubMed/MEDLINE, Embase, Web of Science, Scopus, and the Cochrane Library) initially yielded 629 records. After the removal of 416 duplicate records, 213 unique records remained for title and abstract screening. Following this initial screening, 163 records were excluded as irrelevant to the review question. The full texts of the remaining 50 articles were sought for retrieval, of which three articles could not be obtained. The 47 retrieved full-text articles were assessed for eligibility against the predefined inclusion criteria. Of these, 36 articles were excluded for the following reasons: 13 did not evaluate SGAs as augmentation therapy, nine articles lacked relevant efficacy or safety outcome measures, and 14 were case reports, reviews, or non-randomized study designs. Ultimately, 11 studies [12-22] met all inclusion criteria and were included in the systematic review. The selection process is detailed in the PRISMA flow diagram (Figure 1).

Characteristics of Included Studies

The characteristics of these studies [12-22] are summarized in Table 1. These trials were published between 2016 and 2025, encompassed multinational study populations, and primarily featured double-blind, placebo-controlled, parallel-group designs. Sample sizes ranged from 72 to 812 participants. The definition of TRD was generally consistent, typically requiring an inadequate response (often defined as <50% symptom reduction) to one to three adequate ADTs in the current major depressive episode. The SGAs were used as augmentation to a stable background antidepressant regimen, most commonly selective serotonin reuptake inhibitors or serotonin-norepinephrine reuptake inhibitors. Follow-up durations for the acute efficacy phase varied from 6 to 8 weeks, with one maintenance study extending to 26 weeks [12].

**Table 1:** Characteristics of Included Studies Evaluating Second-Generation Antipsychotic Augmentation in Treatment-Resistant Depression TRD: Treatment-resistant depression; SGA: second-generation antipsychotic; SSRI: selective serotonin reuptake inhibitor; SNRI: serotonin-norepinephrine reuptake inhibitor; MADRS: Montgomery-Asberg Depression Rating Scale; ADT: antidepressant trial; MDD: major depressive disorder

Author (Year)	Country	Study Design	Sample Size (n)	Mean Age (years)	Female % (n)	TRD Definition	Background Antidepressant(s)	SGA Used (Dose Range)	Comparator	Follow-up Duration (weeks)	Primary Outcome Measure
McIntyre et al., [12] (2025)	USA/Poland/Germany	DB RCT (withdrawal)	489	42.3 ± 13.1	63.9%(*734)*	Non-response to 2–3 ADs	SSRI/SNRI	Brexpiprazole (2–3 mg)	Placebo	26	Time to relapse
Kato et al., [13] (2024)	Japan	RCT, DB, PC, multicenter	740	40.4 ± 10.7	44.2% ( 218)	Nonresponse to 1–3 ADTs; <50% HAM-D-17 improvement after 8-wk ADT	SSRI/SNRI	Brexpiprazole 1–2 mg/day	Placebo	6 wk	ΔMADRS
Sachs et al., [14] (2023)	USA, Europe	RCT, DB, PC, Phase 3	759	44.0 ± 13.5	73.6% ( 371)	<50% response to 1–3 AD trials	Stable AD (≥6 wks)	Cariprazine (1.5–3 mg/d)	Placebo + AD	6 wks	MADRS change
Riesenberg et al., [15] (2023)	USA, Canada, Europe	RCT, DB, PC	750	46.2 ±12.1	76.4% (191)	Nonresponse to 1–3 ADTs	Stable ADT	Cariprazine (1.5–3 mg/d)	Placebo + ADT	6	MADRS change
Hobart et al., [16] (2018)	USA, RU, PL, FR, RS, DE, CA	Multicenter DB-RCT	503	43.6 ± 11.5	65.0% ( 128)	<50% response to 1–3 ADTs	SSRI/SNRI	Brexpiprazole (2–3 mg); Quetiapine XR (150–300 mg)	Placebo adj.	6	Δ MADRS
Hobart et al., [17] (2018)	US, DE, PL, SK, HU	RCT, DB, PC	394	44.6 ± 11.6	Not listed	<50% response to 1–3 ADTs	Open-label	Brexpiprazole 2 mg	Placebo	6	MADRS Δ
Durgam et al., [18] (2016)	US, EU	RCT, DB, PC, PG	812	43.5 ± 11.9	58.9% ( 358)	≥1 AD failure ≥6w	Ser, Cit, Esc, Ven, Dul	Cariprazine 1–2, 2–4.5 mg/d	Placebo	8	MADRS Δ
Earley et al., [19] (2018)	USA	R, DB, PC, flex-dose	527	44.0 ± 11.5	64.8% ( 173)	1–2 failed ADTS	Bupropion, citalopram, desvenlafaxine, duloxetine, escitalopram, fluoxetine, sertraline, venlafaxine, paroxetine, vilazodone	Cariprazine 1.5–4.5	Placebo	8	Δ MADRS
Fava et al., [20] (2018)	USA	Randomized, double-blind, placebo-controlled, parallel-group	231 (double-blind ITT)	44.8 ± 11.1	72.1% ( 88)	Failed 1–2 adequate ADTs in current MDD episode	Citalopram, Duloxetine, Escitalopram, Sertraline, Venlafaxine ER	Cariprazine 0.1–0.3 mg/day & 1.0–2.0 mg/day	Placebo + ADT	8	MADRS
Moica et al., [21] (2018)	Romania	Prospective, randomized, open-label	72 (36 per group)	39.8 ± 6.9	72.2% ( 26)	Inadequate response to ≥4–6 weeks of duloxetine monotherapy (HAM-D17 ≥14)	Duloxetine 60 mg/day	Quetiapine XR 150 mg/day	Continuation of duloxetine monotherapy	8	HAM-D17 (Hamilton Rating Scale for Depression)
Kamijima et al., [22] (2018)	Japan, Korea, Malaysia, Taiwan, Australia	RCT, DB, PC	412 (ASC=209, PSC=203)	38.7 ± 9.8	36.4% (76)	<50% HAM-D17 reduction after 1–3 ADT	Sertraline 25–100 mg	Aripiprazole 3–12 mg + Sertraline 100 mg	Placebo + Sertraline 100 mg	6	ΔMADRS (LOCF)

The comparative efficacy and safety outcomes from the included studies are detailed in Table 2. The primary outcome measure across most trials was the change from baseline in the Montgomery-Asberg Depression Rating Scale (MADRS) score.

**Table 2:** Comparative Efficacy and Safety Outcomes of Second-Generation Antipsychotics as Augmentation Therapy in Adult Treatment-Resistant Depression AE: Adverse event; LSMD: least squares mean difference; MADRS: Montgomery-Asberg Depression Rating Scale; URTI: upper respiratory tract infection

Author (Year)	Change in Depression Score (Mean ± SD/MD)	Response Rate (%)	Remission Rate (%)	Effect Size (SMD or RR, 95% CI)	Time to Response (weeks)	Discontinuation Due to AEs (%)	Most Common Adverse Events
McIntyre et al., [12] (2025)	−17.7 ± 9.1	67.6	72.1	NR	6–8 wks	7.1	Headache, akathisia, somnolence, insomnia, weight gain
Kato et al., [13] (2024)	LSM −8.5 (1 mg), −8.2 (2 mg) vs −6.7; MD: −1.7 (1 mg), −1.4 (2 mg)	25.4% (1 mg), 24.5% (2 mg) vs 18.9%	17.7% (1 mg), 17.6% (2 mg) vs 13.6%	MD −1.7 (−3.0 to −0.4); −1.4 (−2.7 to −0.1)	Separation by Week 2	0.8% (1 mg), 7.3% (2 mg)	Akathisia, weight gain, nasopharyngitis, tremor, hyperprolactinemia
Sachs et al., [14] (2023)	MADRS −14.1 / −13.1 vs −11.5 placebo	44.0/39.3	25.2/16.7	LSMD −2.5 / −1.5 (−4.2 to −0.9 / −3.2 to 0.1)	Week 2 (1.5 mg)	1.2 / 7.1	Akathisia, nausea
Riesenberg et al., [15] (2023)	−13.8 to −14.8; LSMD vs placebo: −0.4 to −1.4	No significant difference vs placebo	No significant difference vs placebo	Not significant; early MADRS improvement favored cariprazine	NR	0.8–2.4%	Akathisia, insomnia
Hobart et al., [16] (2018)	6.0	10.5	6.8	LSMD 1.48 (0.39–2.56); RR 1.49	6	1.0	Akathisia, somnolence, headache
Hobart et al., [17] (2018)	−2.30	NR	NR	LSMD −2.30 (−3.97, −0.62)	6	2.1	Akathisia, restlessness, URTI, weight gain
Durgam et al., [18] (2016)	–0.9 to –2.2	↑ vs placebo	NS	LSMD –0.9 to –2.2	2–3	0.4–4.8	Akathisia, insomnia, nausea
Earley et al., [19] (2018)	−7.2 vs −6.5	28.1 vs 27.5	24.4 vs 19.1	LSMD −0.7, 95% CI −1.7 to 0.4, P >0.05	8	8.6	Akathisia, restlessness, insomnia, headache, nausea
Fava et al., [20] (2018)	−7.5 / −0.5 vs placebo (0.1–0.3 mg), −9.8 / −1.8 vs placebo (1–2 mg)	22.4–27.4	19.8–30.3	OR 1.2–1.5 vs placebo	NR	1.3–2.7	Headache, restlessness, fatigue, insomnia, dry mouth, dizziness, constipation, increased appetite, nausea
Moica et al., [21] (2018)	Quetiapine XR 150 mg + Duloxetine	Baseline 22.17 ± 3.23 → Week 8 8.58 ± 3.74	NR	NR	NR	8	NR
Kamijima et al., [22] (2018)	−9.2 ± 0.5 (vs −7.2 ± 0.5 PSC)	37.5	29.3	Response OR 1.73 (1.14–2.65), Remission OR 1.65 (1.05–2.61)	Improvement from week 1	2.9	Akathisia, insomnia, nausea, headache

Efficacy Outcomes

Overall, the augmentation of antidepressants with SGAs demonstrated superior efficacy over placebo in reducing depressive symptoms in most studies. Brexpiprazole showed consistent benefits. A large, multinational withdrawal study by McIntyre et al. [12] reported a mean MADRS reduction of -17.7 and a remission rate of 72.1% during the open-label lead-in phase, with adjunctive brexpiprazole significantly delaying relapse compared to placebo over 26 weeks. Kato et al. [13] found both 1 mg and 2 mg doses of brexpiprazole to be superior to placebo in Japanese patients, with significant differences in MADRS change (MD: -1.7 and -1.4, respectively) and higher response rates (approx. 25% vs. 18.9%) at six weeks. Similarly, two studies by Hobart et al. [16,17] supported the efficacy of flexibly dosed and fixed-dose (2 mg) brexpiprazole, showing significant least squares mean differences (LSMDs) in the MADRS score compared to placebo.

Cariprazine augmentation also showed efficacy, though results were more variable. Sachs et al. [14] and Durgam et al. [18] reported statistically significant separation from placebo on the MADRS as early as week 2-3, with LSMDs ranging from -0.9 to -2.5. Conversely, Riesenberg et al. [15] and Earley et al. [19] found that while cariprazine showed numerical improvement, the difference versus placebo was not statistically significant for the primary endpoint in their respective trials. Fava et al. [20] indicated a dose-dependent effect, with the higher dose range (1-2 mg/day) showing a greater reduction in MADRS scores (-9.8 vs. -7.5 for low dose) compared to placebo.

Regarding other SGAs, Kamijima et al. [22] demonstrated that the combination of aripiprazole and sertraline was significantly more effective than placebo/sertraline, with a greater mean MADRS reduction (-9.2 vs. -7.2) and higher response (OR 1.73) and remission (OR 1.65) rates. Moica et al. [21] reported that adjunctive quetiapine XR (150 mg/day) added to duloxetine resulted in a substantial reduction in Hamilton Rating Scale for Depression (HAM-D17) scores from 22.17 to 8.58 over 8 weeks.

Safety and Tolerability Outcomes

The tolerability profile of SGA augmentation was characterized by higher rates of specific adverse events compared to placebo, though discontinuation rates due to adverse events (AEs) were generally low (ranging from 0.4% to 8.6% across studies). Akathisia was the most frequently reported adverse event associated with brexpiprazole, cariprazine, and aripiprazole [12-20,22]. Other common AEs included insomnia, restlessness, headache, nausea, and somnolence. Weight gain was noted with brexpiprazole and aripiprazole [12,13,17,22]. Quetiapine XR was associated with a discontinuation rate due to AEs of 8%, though specific AEs were not detailed [21].

Risk of Bias

Ten of the eleven RCTs were judged to have a low overall risk of bias [12-20,22]. These studies demonstrated robust methodology, featuring explicit descriptions of randomization, double-blinding, and placebo control; utilization of intention-to-treat analyses; low and balanced attrition rates; and pre-specified, clearly reported primary efficacy outcomes. In contrast, one open-label trial by Moica et al. [21] was assessed as having some concerns due to its design, which introduced potential for performance and detection bias, particularly regarding the administration of the intervention and the assessment of the outcome. Consequently, the findings of this review are primarily supported by evidence from studies with a low risk of bias (Table 3).

**Table 3:** Risk of Bias Assessment Using the Cochrane RoB 2 Tool RoB: Risk of Bias

Study (Year)	Randomization Process	Deviations from Intended Interventions	Missing Outcome Data	Measurement of the Outcome	Selection of Reported Result	Overall Risk of Bias
McIntyre et al., [12] (2025)	Low	Low	Low	Low	Low	Low
Kato et al., [13] (2024)	Low	Low	Low	Low	Low	Low
Sachs et al., [14] (2023)	Low	Low	Low	Low	Low	Low
Riesenberg et al., [15] (2023)	Low	Low	Low	Low	Low	Low
Hobart et al., [16] (2018)	Low	Low	Low	Low	Low	Low
Hobart et al., [17] (2018)	Low	Low	Low	Low	Low	Low
Durgam et al., [18] (2016)	Low	Low	Low	Low	Low	Low
Earley et al., [19] (2018)	Low	Low	Low	Low	Low	Low
Fava et al., [20] (2018)	Low	Low	Low	Low	Low	Low
Moica et al., [21] (2018)	Some concerns	Some concerns	Low	Some concerns	Some concerns	Some concerns
Kamijima et al., [22] (2018)	Low	Low	Low	Low	Low	Low

Discussion

Main Findings

This systematic review synthesized evidence from 11 RCTs evaluating the augmentative efficacy and safety of SGAs in adult patients with TRD. The collective findings indicate that augmentation with SGAs, specifically brexpiprazole, cariprazine, aripiprazole, and quetiapine XR, generally confers a statistically significant and clinically meaningful advantage over placebo in reducing depressive symptoms, with a consistent signal of early onset of action often observed within two to three weeks. The efficacy appears most robust and consistent for brexpiprazole and the aripiprazole-sertraline combination, while the evidence for cariprazine, though largely positive, shows some heterogeneity in outcome significance. The therapeutic benefit, however, is counterbalanced by a distinct and predictable adverse event profile, predominantly characterized by akathisia, which emerges as a class-wide concern alongside other metabolic and neuropsychiatric side effects.

Agent-Specific Efficacy Profiles

The efficacy results for brexpiprazole are particularly compelling. Four of the included studies collectively demonstrate their effectiveness across diverse populations and study designs, including maintenance therapy [12,13,16,17]. The significant delay in relapse shown in the withdrawal study by McIntyre et al. is a critical finding, suggesting that brexpiprazole augmentation may provide sustained benefit beyond acute symptom reduction, a key consideration in a chronic and relapsing condition like TRD [12]. This aligns with and extends the findings from earlier pivotal trials of brexpiprazole, such as those summarized by Thase et al. [23] in a pooled analysis, which established its efficacy and tolerability in MDD with inadequate response to antidepressants. Our review confirms this profile and incorporates more recent and geographically diverse data, reinforcing brexpiprazole's position as a well-evidenced augmentation strategy. The consistent separation from placebo by week two, as seen in several trials, is a clinically valuable attribute, potentially reducing the prolonged suffering associated with sequential medication trials.

The evidence for cariprazine augmentation presents a more nuanced picture. While trials by Sachs et al. and Durgam et al. reported clear efficacy with early onset [14,18], the studies by Riesenberg et al. and Earley et al. failed to find a statistically significant difference on the primary endpoint, despite numerical trends favoring cariprazine [15,19]. This heterogeneity may be attributable to subtle differences in study design, patient population, or dosing regimens. For instance, Fava et al. suggested a dose-response relationship, with the 1-2 mg/day range showing greater effect sizes than lower doses [20]. This variability mirrors discussions in the broader literature on cariprazine in MDD. A meta-analysis by Undurraga et al. [24] concluded that cariprazine was efficacious for adjunctive treatment of MDD, but also noted variability in effect sizes across trials and highlighted dosing as a moderating factor. Our findings are consistent with this appraisal, underscoring that while cariprazine is an effective option, its application may require careful dose optimization.

The result from Kamijima et al. on the aripiprazole-sertraline combination demonstrates significant efficacy, with odds ratios for response and remission exceeding 1.6 [22]. This supports the established role of aripiprazole, which has the longest history and strongest regulatory backing for augmentation in TRD among SGAs. Our finding corroborates the extensive body of evidence for aripiprazole, including the seminal STARD trial sequelae and numerous meta-analyses. For example, a network meta-analysis by Zhou et al. [25] ranked aripiprazole among the most efficacious augmenting agents for TRD. The study by Moica et al. on quetiapine XR, though limited by its open-label design and smaller sample size, reported a dramatic reduction in HAM-D17 scores [21]. This adds to the existing, though somewhat less consistent, literature on quetiapine. A Cochrane review by Komossa et al. [26] found quetiapine effective as an add-on treatment but with a less favorable tolerability profile due to sedation and metabolic effects. Our review suggests it remains a viable option, particularly when sedation is clinically acceptable or desired.

Safety and Tolerability Implications

The comparative analysis of safety and tolerability is perhaps where the most consistent class effect emerges. Akathisia was the predominant adverse event across studies of brexpiprazole, cariprazine, and aripiprazole, affecting a substantial minority of patients and representing a leading cause of discomfort and potential discontinuation. This finding is not novel but is powerfully reinforced by the concordant data across eleven trials. It echoes the concerns raised in previous reviews, such as that by Cantu et al. [27], which critically examined the efficacy and side effects of atypical antipsychotics for depression and highlighted akathisia as a common and distressing problem. Other frequent side effects like insomnia, restlessness, headache, and weight gain further delineate the risk-benefit calculus. The generally low discontinuation rates due to adverse events (mostly under 10%) indicate that these side effects are often manageable in a clinical trial setting with proactive monitoring, but they undeniably impact the acceptability and quality of life for patients. The weight gain and metabolic implications, particularly noted with brexpiprazole and aripiprazole, necessitate long-term monitoring, aligning with concerns raised in guidelines for the use of antipsychotics in mood disorders.

Contextualization Within the Broader TRD Treatment Landscape

When contextualized within the broader landscape of TRD treatment, the findings of this review affirm the position of SGA augmentation as a cornerstone of evidence-based practice. Our results are congruent with major treatment guidelines, such as those from the Canadian Network for Mood and Anxiety Treatments (CANMAT), which recommend several SGAs (including aripiprazole, quetiapine, and brexpiprazole) as first-line augmentation options for TRD [28]. The efficacy demonstrated here is comparable or superior to other augmentation strategies like lithium or thyroid hormone, which have more limited evidence bases and their own unique tolerability challenges. For instance, a meta-analysis by Nelson & Papakostas [29] found that augmentation with atypical antipsychotics yielded a significantly higher response rate compared to placebo, with a number needed to treat comparable to other strategies but with a different side effect profile. The early onset of action observed for several SGAs is a distinct advantage over some alternatives, potentially offering a quicker exit from a depressive episode.

Demographic Generalizability and Critical Perspectives on Trial Diversity

A critical consideration for interpreting the findings of this review is the demographic composition of the included trial populations. The majority of participants across the 11 RCTs were predominantly Caucasian, with limited representation of ethnic and racial minorities. This lack of diversity is not unique to these trials but reflects a well-documented and persistent problem across psychiatric clinical research more broadly. The underrepresentation of minority populations significantly limits the external validity and generalizability of the efficacy and safety data to non-Caucasian populations. This is particularly relevant for the metabolic safety of SGAs, as pharmacogenomic variations across ethnic groups can influence drug metabolism, receptor sensitivity, and susceptibility to adverse effects such as weight gain, dyslipidemia, and glucose dysregulation. For instance, genetic polymorphisms in cytochrome P450 enzymes and serotonin and dopamine receptor genes, which may vary in frequency across ancestral populations, could alter both therapeutic response and side effect burden. Consequently, the metabolic risk profiles described in this review, derived from largely homogeneous samples, may not fully capture the safety implications for more diverse patient populations encountered in routine clinical practice. Future trials must prioritize the intentional recruitment of ethnically and racially diverse samples and stratify analyses by these variables, to ensure that treatment recommendations are truly evidence-based for all patients.

Risk of Bias and Internal Validity

However, the discussion must also consider the implications of the risk of bias assessment. The fact that 10 of the 11 included studies were judged to have a low overall risk of bias strengthens the internal validity of our conclusions regarding efficacy and safety for brexpiprazole, cariprazine, and aripiprazole. These were predominantly large, rigorously conducted, pharmaceutical industry-sponsored phase 3 trials designed for regulatory approval. While this ensures high methodological quality, it may also limit generalizability. Trial participants are often highly selected, with strict inclusion/exclusion criteria that may exclude patients with significant comorbidities, suicidality, or more complex treatment histories commonly seen in real-world practice. The sole study with some concerns, the study by Moica et al., reminds us that open-label designs, while pragmatic, introduce potential bias that can inflate perceived efficacy [21]. Therefore, while the efficacy data are robust, their absolute magnitude may be somewhat attenuated in routine clinical settings.

Evidence Gaps and Future Research Directions

Furthermore, our review highlights several critical evidence gaps that future research must address. First, there is a striking absence of direct head-to-head comparisons between different SGAs for augmentation. Clinicians and guideline developers are left to infer relative efficacy and tolerability from indirect comparisons across separate placebo-controlled trials, which is methodologically inferior. A randomized trial directly comparing, for example, brexpiprazole to aripiprazole would provide invaluable practical guidance. Second, the long-term efficacy and safety data beyond 6-8 months are sparse, with the maintenance study by McIntyre et al. being a notable exception [12]. TRD is a chronic condition, and understanding the sustainability of benefit and the long-term metabolic and neurological consequences of years of SGA augmentation is paramount. Third, there is insufficient evidence on predictors of response. Identifying clinical or biological markers that could predict which patients are most likely to benefit from a particular SGA would personalize treatment and improve outcomes. Finally, the economic impact and cost-effectiveness of these often expensive branded medications compared to other augmentation strategies require thorough investigation to inform healthcare policy.

Limitations

This systematic review has several limitations. First, the search was limited to published studies in selected databases, raising the possibility of publication bias, where negative or equivocal trials may be less likely to be published. Although the included studies themselves had low risk of bias, the synthesis at the review level may be affected by this. Second, significant clinical heterogeneity existed among the studies in terms of the precise definition of TRD, the background antidepressants permitted, the dosing regimens of the SGAs, and the study durations. This heterogeneity precluded a formal meta-analysis with pooled effect estimates, which would have provided a more quantitative summary of the evidence. Instead, our synthesis is narrative and qualitative. Third, as noted, the generalizability of findings from stringent RCTs to the broader, more comorbid population seen in clinical practice is uncertain. Fourth, safety data were reported inconsistently across studies, often focusing on common short-term adverse events, which limited our ability to fully characterize and compare the tolerability profiles, especially regarding rare but serious long-term risks. Finally, this systematic review was not prospectively registered with PROSPERO, which should be considered when interpreting the findings.

## Conclusions

This systematic review provides robust and current evidence supporting the use of SGAs, particularly brexpiprazole, cariprazine, aripiprazole, and quetiapine XR, as effective augmentation strategies for adults with TRD. These agents offer a clinically significant reduction in depressive symptoms, with an encouraging trend towards early onset of action. Brexpiprazole currently has the most consistent and extensive evidence base, including maintenance data. However, this therapeutic benefit is uniformly tempered by a substantial risk of AEs, most notably akathisia, which necessitates careful patient selection, thorough informed consent, and proactive management. The findings are largely congruent with and reinforce existing treatment guidelines. Future research should prioritize direct comparative effectiveness trials, long-term outcome studies, and investigations into predictive biomarkers to advance the field toward more personalized and sustainable management of this challenging condition.

## Appendices

**Table 4:** Search Strategies for Databases

Database	Search String
PubMed/MEDLINE	("Depressive Disorder, Treatment-Resistant"[Mesh] OR "treatment-resistant depression"[tiab] OR "treatment refractory depression"[tiab] OR "refractory depression"[tiab]) AND ("Antipsychotic Agents"[Mesh] OR "second-generation antipsychotic"[tiab] OR "atypical antipsychotic"[tiab] OR brexpiprazole[tiab] OR cariprazine[tiab] OR aripiprazole[tiab] OR quetiapine[tiab] OR olanzapine[tiab] OR risperidone[tiab] OR ziprasidone[tiab] OR lurasidone[tiab]) AND ("randomized controlled trial"[pt] OR "controlled clinical trial"[pt] OR randomized[tiab] OR placebo[tiab] OR randomly[tiab] OR trial[tiab]) NOT ("animals"[mh] NOT "humans"[mh])
Embase	('treatment resistant depression'/exp OR 'treatment-resistant depression':ti,ab OR 'treatment refractory depression':ti,ab OR 'refractory depression':ti,ab) AND ('atypical antipsychotic agent'/exp OR 'second-generation antipsychotic':ti,ab OR 'atypical antipsychotic':ti,ab OR brexpiprazole:ti,ab OR cariprazine:ti,ab OR aripiprazole:ti,ab OR quetiapine:ti,ab OR olanzapine:ti,ab OR risperidone:ti,ab OR ziprasidone:ti,ab OR lurasidone:ti,ab) AND ('randomized controlled trial'/exp OR 'crossover procedure'/exp OR 'double blind procedure'/exp OR 'single blind procedure'/exp OR random*:ti,ab OR placebo:ti,ab OR 'clinical trial':ti,ab) AND [humans]/lim AND (2016-2025)/py
Web of Science	(TI=("treatment-resistant depression" OR "treatment refractory depression" OR "refractory depression") OR AB=("treatment-resistant depression" OR "treatment refractory depression" OR "refractory depression")) AND (TI=("second-generation antipsychotic" OR "atypical antipsychotic" OR brexpiprazole OR cariprazine OR aripiprazole OR quetiapine OR olanzapine OR risperidone OR ziprasidone OR lurasidone) OR AB=("second-generation antipsychotic" OR "atypical antipsychotic" OR brexpiprazole OR cariprazine OR aripiprazole OR quetiapine OR olanzapine OR risperidone OR ziprasidone OR lurasidone)) AND (TI=(randomized OR randomised OR placebo OR "clinical trial") OR AB=(randomized OR randomised OR placebo OR "clinical trial"))
Scopus	TITLE-ABS-KEY("treatment-resistant depression" OR "treatment refractory depression" OR "refractory depression") AND TITLE-ABS-KEY("second-generation antipsychotic" OR "atypical antipsychotic" OR brexpiprazole OR cariprazine OR aripiprazole OR quetiapine OR olanzapine OR risperidone OR ziprasidone OR lurasidone) AND TITLE-ABS-KEY(randomized OR randomised OR placebo OR "clinical trial") AND PUBYEAR > 2015
Cochrane Library	("treatment-resistant depression" OR "treatment refractory depression" OR "refractory depression"):ti,ab,kw AND ("second-generation antipsychotic" OR "atypical antipsychotic" OR brexpiprazole OR cariprazine OR aripiprazole OR quetiapine OR olanzapine OR risperidone OR ziprasidone OR lurasidone):ti,ab,kw AND (randomized OR randomised OR placebo OR "clinical trial"):ti,ab,kw (with Cochrane Central Register of Controlled Trials selected)
